# Post-transplant lymphoproliferative disorder presenting as T-prolymphocytic leukemia: a case report

**DOI:** 10.1186/s13256-019-2164-y

**Published:** 2019-07-22

**Authors:** Ganesh Kasinathan, Ahlam Naila Kori, Norasyikin Mohamad Azmie

**Affiliations:** 1 Haematology Unit, Department of Internal Medicine, Tengku Ampuan Afzan Hospital, 25100 Kuantan, Pahang Malaysia; 2 Haematopathology Unit, Department of Pathology, Tengku Ampuan Afzan Hospital, Kuantan, Pahang Malaysia

**Keywords:** Prolymphocytic leukemia, Renal transplant, Tumor lysis, Venetoclax

## Abstract

**Introduction:**

Post-transplant lymphoproliferative disorder is a serious disorder which occurs post hematopoietic stem cell transplant or solid organ transplantation. T-prolymphocytic leukemia is a T cell type monomorphic post-transplant lymphoproliferative disorder which accounts for only 2% of all mature lymphocytic leukemias in adults over the age of 30.

**Case presentation:**

A 59-year-old man of Chinese ethnicity presented to our hematology unit with headache, lethargy, and exertional dyspnea for the past 1 month. He underwent an uneventful cadaveric renal transplant 20 years ago for chronic glomerulonephritis-induced end-stage renal disease. He had been on long-term immunosuppressants since then consisting of orally administered prednisolone 10 mg daily and orally administered cyclosporine A 50 mg twice daily. On examination, he was pale with a palpable liver and spleen. He had a functioning renal graft. Marrow flow cytometry confirmed T-prolymphocytic leukemia with lymphocytes expressing CD2, CD3, CD7, CD52, and TCL-1. His human T-cell lymphotropic virus and Epstein–Barr virus serology and deoxyribonucleic acid (DNA) were negative. He was treated with one cycle of cyclophosphamide, doxorubicin, vincristine, and prednisone chemotherapy to which he failed to respond. In view of his renal allograft, he was not suitable for alemtuzumab due to the risk of nephrotoxicity. He was given orally administered venetoclax but he died on day 17 due to severe auto tumor lysis syndrome.

**Conclusion:**

The place of immunophenotyping in the diagnosis and treatment of this disorder is of significant importance. More research needs to be carried out to further comprehend the pathophysiology and treatment modalities for this disorder.

## Introduction

Post-transplant lymphoproliferative disorder (PTLD) is a serious disorder which occurs post hematopoietic stem cell transplant (HSCT) or solid organ transplantation. The World Health Organization (WHO) 2016 classification system categorizes PTLD into six different categories: infectious mononucleosis PTLD, plasmacytic hyperplasia PTLD, florid follicular hyperplasia PTLD, polymorphic PTLD, monomorphic PTLD arising from B and T/natural killer (NK) T cell types, and classical Hodgkin lymphoma PTLD [1]. Approximately 90–95% of cases of PTLD are attributable to the B cell lineage while the remaining 5–10% are T/NK cell or Hodgkin lymphoma. Epstein–Barr virus (EBV) infection is frequently implicated in the pathogenesis of PTLD as most recipients of a transplant are on chronic immunosuppressants which lead to inhibition of T cell function. The inhibition of T cell function favors the proliferation of EBV. The incidence of PTLD in patients post solid organ transplant and HSCT is approximately 20% and 4%, respectively [2]. The most frequent incidence of PTLD is seen in intestinal and multi-organ transplant recipients (5–20%), followed by heart and lung transplants (2–10%), and then by liver and kidney transplants (1–5%) [2]. Prolymphocytic leukemia (PLL) falls into the monomorphic PTLD subtype. PLL is a rare lymphocytic disorder which accounts for only 2% of all mature lymphocytic leukemias in adults over the age of 30 [3]. It can be further categorized into T and B cell subtypes with differing laboratory and clinical manifestations. T-prolymphocytic leukemia (T-PLL) is an aggressive mature post thymic leukemia with an unfavorable prognosis. It is a very rare disease and was first described in 1973 [4]. Patients diagnosed as having T-PLL typically have systemic manifestations at diagnosis such as hepatosplenomegaly, widespread lymphadenopathies, and skin infiltrates [5].

## Case presentation

A 59-year-old man of Chinese ethnicity presented to our hematology unit with headache, lethargy, and exertional dyspnea for the past 1 month. He underwent an uneventful cadaveric renal transplant 20 years ago for chronic glomerulonephritis-induced end-stage renal disease. He had been on long-term immunosuppressants since then consisting of orally administered prednisolone 10 mg daily and orally administered cyclosporine A 50 mg twice daily. He is married with four children. He had no significant family history. He did not smoke tobacco and did not drink alcohol. He works as a contractor with a construction company.

On examination, he was pale. Cardiovascular and respiratory examinations were unremarkable. He had a palpable liver and spleen of 3 cm. There were no palpable lymph nodes. The renal graft was palpable and of normal size.

His complete blood count revealed normochromic normocytic anemia of 7.4 g/dL with leukocytosis of 54 × 10^9^/L (predominantly lymphocytosis) and a normal platelet count of 202 × 10^9^/L. The other laboratory parameters on presentation are tabulated in Table 1.

**Table 1:** Shows the laboratory parameters and their values

Laboratory parameters	Values (unit and normal range)
Hemoglobin	7.4 (13.5–16 g/dL)
Total white cell count	54 (4–10 × 10^9^/L)
Absolute lymphocyte count	44 (1–3 × 10^9^/L)
Platelet	202 (150–400 × 10^9^/L)
Creatinine	90 (40–100 umol/L)
Lactate dehydrogenase (LDH)	472 (90–180 U/L)
Serum uric acid	205 (68–117 umol/L)
Serum calcium	2.3 (2.2–2.6 mmol/L)
Serum phosphate	1.2 (0.8–1.5 mmol/L)
Aspartate aminotransferase	40 (0–40 U/L)
Alanine aminotransferase	38 (0–40 U/L)
Erythrocyte sedimentation rate (ESR)	80 (0–20 mm/hour)
C-reactive protein (CRP)	25 (0–5 mg/L)
HIV 1 and 2 serology	Non-reactive
Epstein–Barr virus serology and PCR	Not detected
Cytomegalovirus serology and PCR	Not detected
Human T-cell lymphotropic virus types I and II	Not detected

*PCR* polymerase chain reaction

**Fig. 1:**
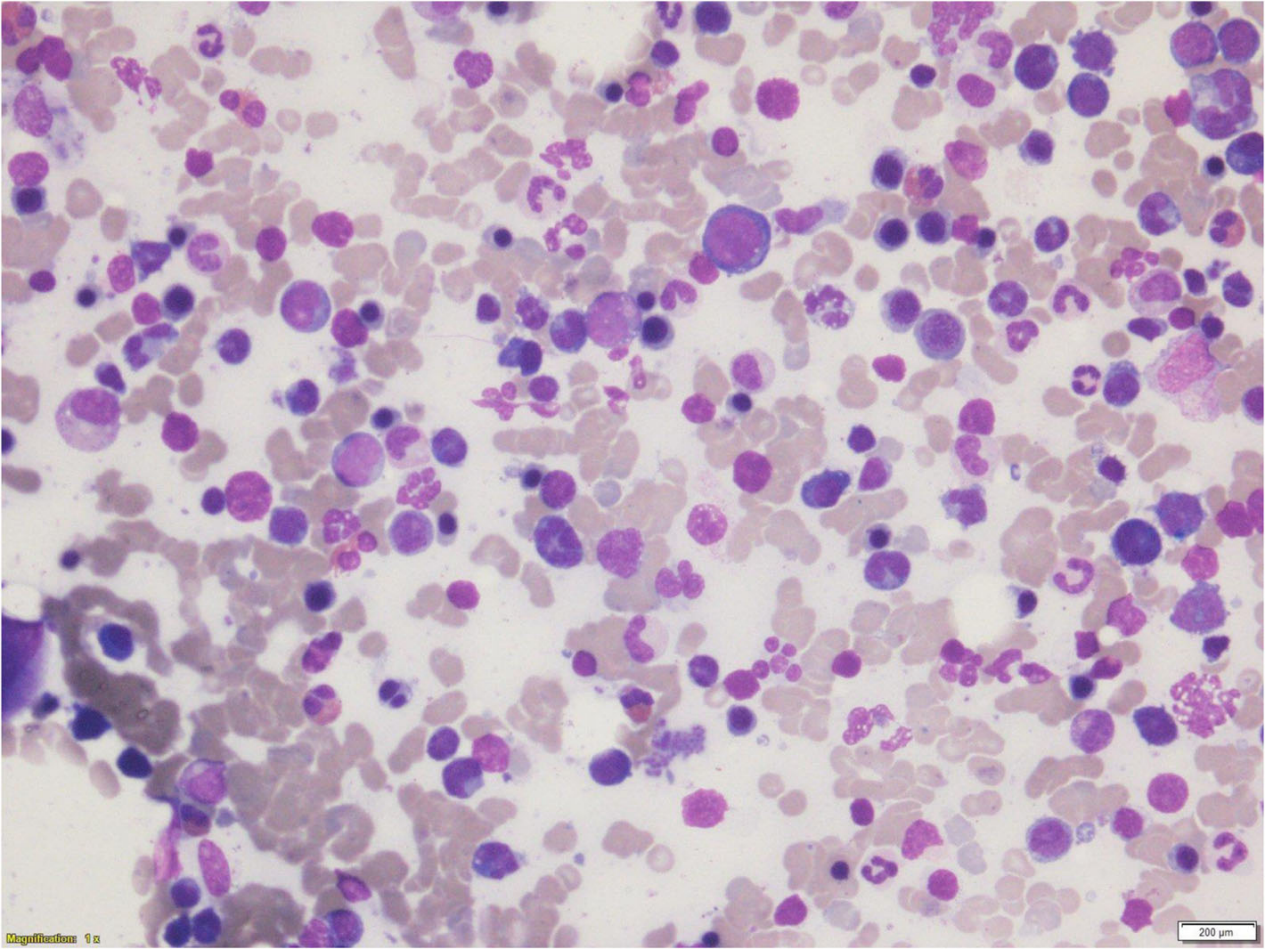
The bone marrow smear shows a markedly hypercellular marrow with diffuse infiltrates of abnormal lymphoid cells which are pleomorphic, moderate to large in size, contain multiple coarse chromocenters in convoluted nuclei with irregular nuclear outline and scanty cytoplasm

**Fig. 2:**
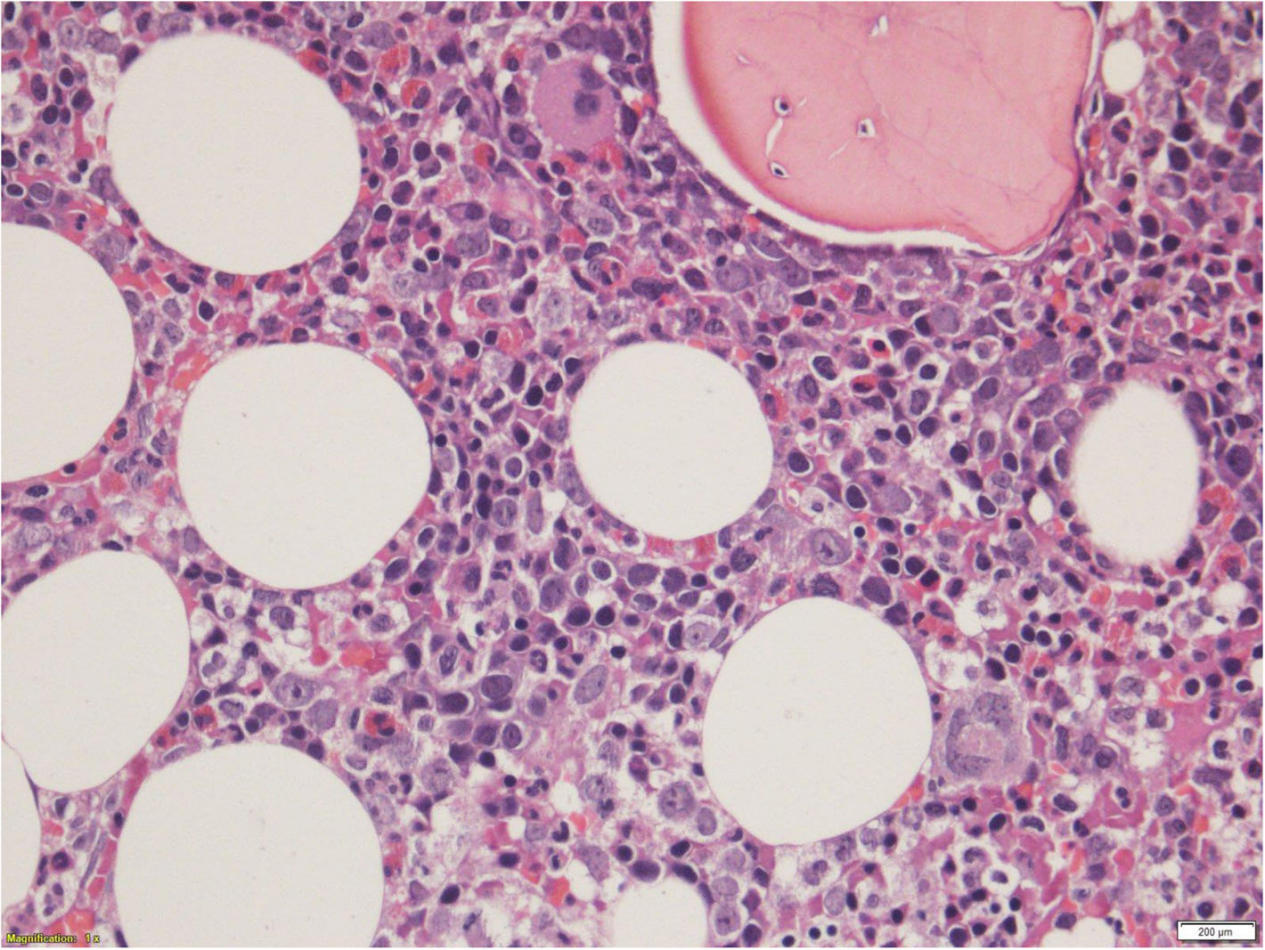
Bone marrow trephine biopsy shows infiltrate which is in an interstitial distribution

A peripheral blood film revealed a leukoerythroblastic picture with presence of abnormal mononuclear cells. A bone marrow smear (Fig. 1) showed a markedly hypercellular marrow with diffuse infiltrates of abnormal lymphoid cells which were pleomorphic, moderate to large in size, and contained multiple coarse chromocenters in convoluted nuclei with irregular nuclear outline and scanty cytoplasm. The trephine (Fig. 2) was consistent with T-PLL.

Marrow flow cytometry analysis showed 25% abnormal population of cells expressing CD2, CD3, CD7, CD52, and TCL-1. The cell population was negative for CD4, CD8, CD25, CD56, CD57, CD94, and cMPO.

In view of the bone marrow and flow cytometry findings, he was diagnosed as having T-PLL which is an aggressive form of a lymphoproliferative disorder.

He was treated with one cycle of cyclophosphamide, doxorubicin, vincristine, and prednisone (CHOP) chemotherapy to which he failed to respond.

He was then started on orally administered venetoclax daily. Since he has a renal graft, the option of anti-CD52 (alemtuzumab) therapy was not suitable due to the risk of cytomegalovirus (CMV) activation and the renal toxicity of ganciclovir.

Subsequently, on day 15 of orally administered venetoclax, he developed severe auto tumor lysis syndrome which required mechanical ventilation and continuous veno-venous hemodialysis. His white cell count progressed to 174 × 10^9^/L, predominantly lymphocytosis. He died 2 days later at our intensive care unit.

## Discussion

PTLD is known to occur post solid organ transplant or HSCT. It was first described in recipients of renal transplant in the year of 1968 by Doak *et al.* and the term PTLD was first coined in 1984 by Starzl *et al*. [6]. The higher incidence of PTLD seen in haploidentical stem cell transplant and solid organ transplant involving intestine, multiple organs, heart, and lung could be explained by histo-incompatibility and the amount of lymphoid tissue present in the grafts. Grafts containing a substantial amount of lymphoid tissues, such as the small intestine, can result in transfer of potentially EBV-infected donor lymphocytes which may drive the development of PTLD [7].

In our case, we described a very aggressive course of T-PLL which occurred as a T cell type monomorphic PTLD in a patient who was on chronic usage of immunosuppressants post renal transplant. T-PLL primarily affects older individuals with an average age of 65 years at presentation with a slight male predominance [8]. In T-PLL, there is usually marked lymphocytosis in the peripheral blood which is often more than 100 × 10^9^/L with most of them being prolymphocytes. Renal function, including calcium levels, is normal; liver function tests may show a mild impairment. Hyperuricemia and a slightly raised lactate dehydrogenase (LDH) are common features [9]. Frequently, human T-cell lymphotropic virus (HTLV) types 1 and II serology and polymerase chain reaction (PCR) are negative [9].

T-PLL consists of prolymphocytes which are medium sized and often contain a single nucleoli and basophilic cytoplasm with occasional projections [10]. The nuclei are usually round to oval in shape, with some patients having cells with a more irregular nuclear outline that are similar to the cerebriform nuclear shape seen in Sézary syndrome [10]. Bone marrow trephine biopsies usually reveal infiltrate which is interstitial and nodular with an intertrabecular distribution [11].

On immunophenotyping, the cells show positivity for CD2, CD3, CD5, CD7, and CD 52 staining. The CD7 intensity is strong in contrast to other mature T cell malignancies, where this marker may be weak or negative. CD4 and CD8 can be co-expressed in 21% of cases and CD8 alone in 13% of cases [12]. The most specific markers for T-PLL by flow cytometry are CD26 and TCL-1 protein expression, which are not detected in the other mature T cell leukemia/lymphomas [13]. The overexpression of the oncogene *TCL1* is useful for detecting residual T-PLL in bone marrow sections after therapy [13].

T-PLL is characterized by complex cytogenetic abnormalities, which may explain the aggressive nature of this disease. Recurrent changes mainly affect chromosomes 14, 8, 11, and X [14].

Abnormalities of chromosome 8 are seen in approximately 75% of patients, including idic(8p11), t(8;8)(p11-12;q12), and trisomy 8 [15].

In 80% of patients, the cytogenetics reveal inversion of chromosome 14 [inv14(q11;q32)] and in 10% translocation t(14)(q11;q32) inducing activation of the *TCL1* oncogene [15]. The expression of the *MTCP1* gene which has homologies with *TCL1* has been reported in rare cases of T-PLL [16]. Activation of *TCL1* appears to be the initiator of T-PLL oncogenesis.

T-PLL is often resistant to conventional chemotherapy. Approximately one third of the patients respond to CHOP chemotherapy. The disease eventually recurs in all of these patients [17]. The prognosis is poor for those with bulky disease but the average survival has increased by 2 years due to the availability of anti-CD52 which is alemtuzumab. The best responses have been seen with alemtuzumab, but responses are still transient and further disease progression is inevitable [18]. Alemtuzumab may result in reactivation of CMV infection and the usage of ganciclovir to treat CMV is potentially nephrotoxic in a patient with a renal graft.

Allogenic stem cell transplantation should be considered in younger patients who have responded to their initial therapy, as this disease tends to progress. There is a possibility of long-term cure by potentially harnessing a graft-versus-leukemia effect [19].

A research group led by hemato-oncologist Philipp Staber, from the Medical University of Vienna’s Division of Hematology and Hemostaseology, and biochemist Stefan Kubicek, from the Research Center for Molecular Medicine, found that venetoclax (BCL-2 inhibitor) is useful in T-PLL. The protein BCL-2 is imperative for the survival of T-PLL cells. Besides that, venetoclax has minimal adverse effects.

## Conclusion

PTLD T-PLL is a very rare disease and it can be easily missed by practicing physicians. The place of immunophenotyping in the diagnosis and treatment of this disorder is of significant importance. More research needs to be carried out to further comprehend the pathophysiology and treatment modalities for this disorder.

## Data Availability

Not applicable.
